# Supplementary material for: Rationalisation of the UK Nutrient Databank for Incorporation in a Web-Based Dietary Recall for Implementation in the UK National Diet and Nutrition Survey Rolling Programme
Source: Nutrients. 2022 Oct 28;14(21):4551. doi: 10.3390/nu14214551 (PMC9658736; doi:10.3390/nu14214551)
Supplement: Supplementary file 1 [file nutrients-14-04551-s001.zip › Supplementary material-File S1.pdf]

File S1. Rationalisation and evaluation tables and figures

Table S1a. Example of fat free yogurts in extensive NDB and food matching decisions.

Food code in extensive NDB	Food name	Consumption 2008-2018 (n)*	Status	Matched food code in extensive NDB
2701	Yogurt, virtually fat free, fruit with artificial sweetener	522	Retain[1]	
2700	Yogurt, virtually fat free, natural, unsweetened	164	Retain[1]	
8990	Yogurt, virtually fat free, fruit, added sugar	67	Retain[1]	
11026	Greek style virtually fat free fruit yogurt e.g. Müller Light	67	Retain[1]	
11249	Yogurt virtually fat free, natural, strained (high protein)	43	Retain[1,2]	
8041	Virtually fat free yogurt with added fibre	211	Represent[3]	2701
11208	Yogurt virtually fat free fruit with sweeteners fortified	28	Represent[3]	2701
9272	Yogurt, virtually fat free, any other flavour, with artificial sweetener	208	Represent[3]	2701
11147	Yogurt virtually fat free, fruit, strained without cream	19	Represent[3]	8990
11310	Muller light fat free yogurt with added calcium	24	Represent[3]	11026
11161	Fat free yogurt containing fruit, Benecol only	0	Exclude[4]	

*Total number of occasions where the food is reported by NDNS respondents between NDNS Years 2008-2018. [1]Retained in rationalised NDB as frequently consumed. [2]Retained in rationalised NDB as nutritionally different to other similar foods. [3]Represented as nutritional composition matched to another similar food, therefore removed from rationalised NDB. [4]Excluded from rationalised NDB as it wasn't frequently consumed.

Table S1b. List of the descriptions of fried bacons reported in NDNS Year 2017 which were ultimately grouped under "bacon fried, homemade" and matched to "Food Code 909, bacon rashers fried lean and fat" in rationalised NDB.

Original recipe names reported by respondents
Back bacon fried in blended vegetable oil
Bacon fried homemade
Bacon fried in butter
Bacon fried in BVO
Bacon fried in crisp n dry
Bacon fried in Frylight
Bacon fried in olive oil
Bacon fried in olive oil homemade
Bacon fried in sunflower oil
Bacon fried in sunflower oil homemade
Bacon fried in veg oil
Bacon fried in veg oil homemade
Bacon medallion fried in sunflower oil
Bacon*cook*rap
Fried bacon
Fried bacon – café
Fried bacon - h/m
Streaky bacon fried in butter
Streaky bacon fried in crisp n dry
Streaky bacon fried in vegetable oil

Table S1c. Matching recipes to single foods for purpose of evaluation exercise, the example "lentil dish"*.

Ingredient food groups[1]	n[2]	Matched food code[3]
Bean-pulses : Savoury sauce	2	FC 1758- Lentils split, boiled
Tap water : Bean-pulses : Tomatoes not raw : Other vegetable : Salad and other vegetable : Other oil : Savoury sauce	2	FC 1761- Lentil curry
Bean-pulses : Tap water : Other vegetable : Savoury sauce : Other oil	1	FC 2469-Lentil soup
Other cereals: Bean-pulses : Polyunsaturated oils : Butter : Savoury sauce	1	FC 2605-Bombay mix
Bean-pulses : Other potato : Other vegetable : Leafy green vegetables : Savoury sauce : Tap water : Salad and other vegetable : Savoury sauce	1	FC 3706-Lentil curry with vegetables

*Name of the recipe given by participant. [1]The food groups that each ingredient in "lentil dish" recipe belongs to. [2]n=number of times recipe consumed in NDNS 2017. [3]Food code in Rationalised NDB.

Table S1d. Differences in the mean daily intake of nutrients from all sources including nutritional supplements in NDNS 2017 respondents (n=1211) aged 1.5-93 years.

Nutrient	Extensive NDB (n=5933)		Rationalised NDB (n=2481)		Diff.[1]	*P-value*	Cohen's *d*
	Mean	SD	Mean	SD			
Vitamin B_1 (mg)	1.67	1.95	1.82	2.21	0.15	<0.01	0.07
Vitamin B_2 (mg)	1.78	2.10	1.83	2.05	0.05	0.12	0.02
Niacin (mg)	31.8	13.9	32.9	14.6	1.1	<0.01	0.08
Vitamin E (mg)	10.8	14.0	11.6	14.6	0.9	<0.01	0.06
Calcium (mg)	795	327	798	325	3	0.36	0.01
Magnesium (mg)	232	92	235	91	3	<0.01	0.03
Zinc (mg)	7.94	4.15	8.12	4.27	0.19	<0.01	0.04
Iron (mg)	10.3	9.7	10.6	10.3	0.27	<0.01	0.03
Potassium (mg)	2462	838	2483	836	21	<0.01	0.03

[1]Difference: Calculated as the difference of the mean intake (rationalised-extensive NDB).

Table S1e. Association between estimates of nutrient intake from all sources including nutritional supplements using the extensive (n=5933) and rationalised (n=2481) NDB.

Nutrient	Proportion in the same tertile (%)	Cohen's *κ*
Vitamin B_1 (mg)	82.3	0.73
Vitamin B_2 (mg)	87.9	0.82
Niacin (mg)	84.2	0.76
Vitamin E (mg)	72.1	0.58
Calcium (mg)	85.6	0.78
Magnesium (mg)	89.0	0.84
Zinc (mg)	86.7	0.80
Iron (mg)	86.4	0.80
Potassium (mg)	89.5	0.84

Figure S1a. Respondents (%) moving categories of nutrient adequacy for total fat recommendation (20-35 %TE) using the extensive and rationalised NDB*.

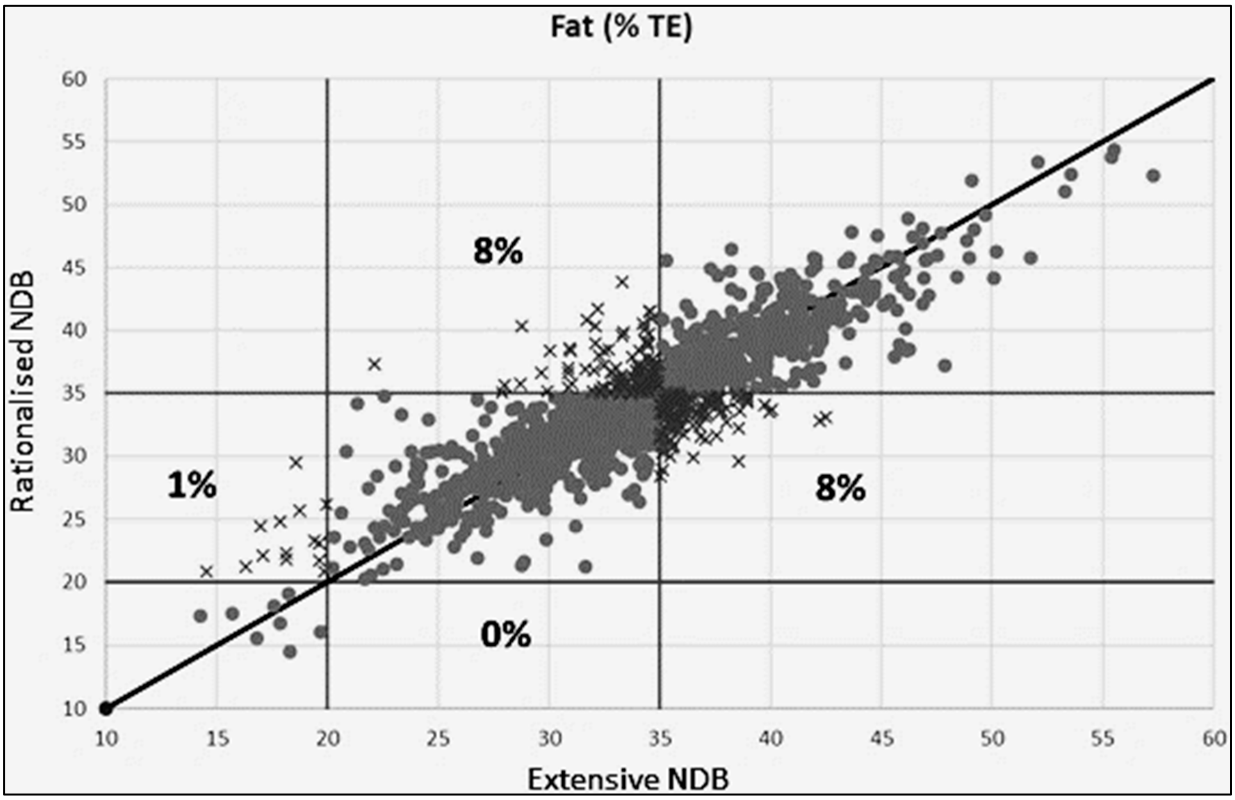

*Dots are respondents who do not move category; crosses are respondents who do move category.
