# Supplementary material for: Rationalisation of the UK Nutrient Databank for Incorporation in a Web-Based Dietary Recall for Implementation in the UK National Diet and Nutrition Survey Rolling Programme
Source: Nutrients. 2022 Oct 28;14(21):4551. doi: 10.3390/nu14214551 (PMC9658736; doi:10.3390/nu14214551)
Supplement: Supplementary file 1 [file nutrients-14-04551-s001.zip › Supplementary material-File S2.pdf]

File S2. Food matching process, yogurt example.

Step 1. Determination of nutrient cut-offs

The nutrient cut-offs were determined by taking the 25% of average Reference Nutrient intakes for 1 to 3-year-olds, the youngest age group in NDNS to retain maximum sensitivity in the food reference database. For example, for protein, the Reference Nutrient Intake is 14.5g/day [1]. The cut-off for protein is 3.6g/100g of edible portion of foods which is 25% of 14.5g/day.

Three key nutrients were determined for each food group by calculating the lowest food amount matching the cut-off for each food group. For example calcium, protein and sugar were key nutrients for the yogurt group which included 58 yogurts and the lowest average amount of yogurt (68-96g) was matching the cut-offs of calcium, protein, and sugar (see Table S2a).

Table S2a. Determining three key nutrients, example yogurt.

Food Group Description	The amount of yogurt matching the cut-offs[1] (n=58)
Energy	285g
Fat	337g
CHO	261g
Sugar	96g
Saturated fat	172g
Protein	86g
Fibre	1479g
Ca	68g
Fe	1091g
Folate	194g
VitC	447g
VitD	1168g
1st lowest amount is met by:	Calcium
2nd lowest amount is met by:	Protein
3rd lowest amount is met by:	Sugar

[1]The nutrient cut-offs were determined by taking the 25% of average Reference Nutrient intakes for 1 to 3-year-olds, the youngest age group in NDNS.

Step 2- For example, for making a decision if the yogurt code 8990 (yogurt, virtually fat free, fruit, added sugar) could be represented by food code 11147 (yogurt virtually fat free, fruit, strained without cream), the composition of the yogurts was listed in Table S2b and the difference between the values was calculated. The average portion size consumption for both yogurts was 122g. The difference was calculated for 122g. The gram difference between food code 8990 and food code 11147 was below 25% cut-offs. Therefore these two codes could be represented by one code and the less consumed food was excluded. This comparison was done using the integrated dietary assessment system, Diet In Nutrients Out (DINO)[2] and the screenshot of its feature used for the comparison of foods in pairs is displayed in Figure S2a.

Table S2b. Comparison of the key nutrient content of two yogurt codes

Key nutrients	Yogurt FC 8990*	Yogurt FC 11147*	25% cut-off	The difference in 122g per average consumed portion size	The difference compared to 25% cut-off
Calcium (mg)	150	112	87.5	42	<cut-off
Protein (g)	4.5	7.7	3.6	3.5	<cut-off
Sugar (g)	14.7	12.4	10.6	2.5	<cut-off

FC: Food code, *in 100g of edible portion of food.

Figure S2a. The screen of Diet In Nutrients Out (DINO) [2] used for the comparison of foods in pairs using the cut-offs, key nutrients and the average amount of foods consumed in NDNS survey year 2017.

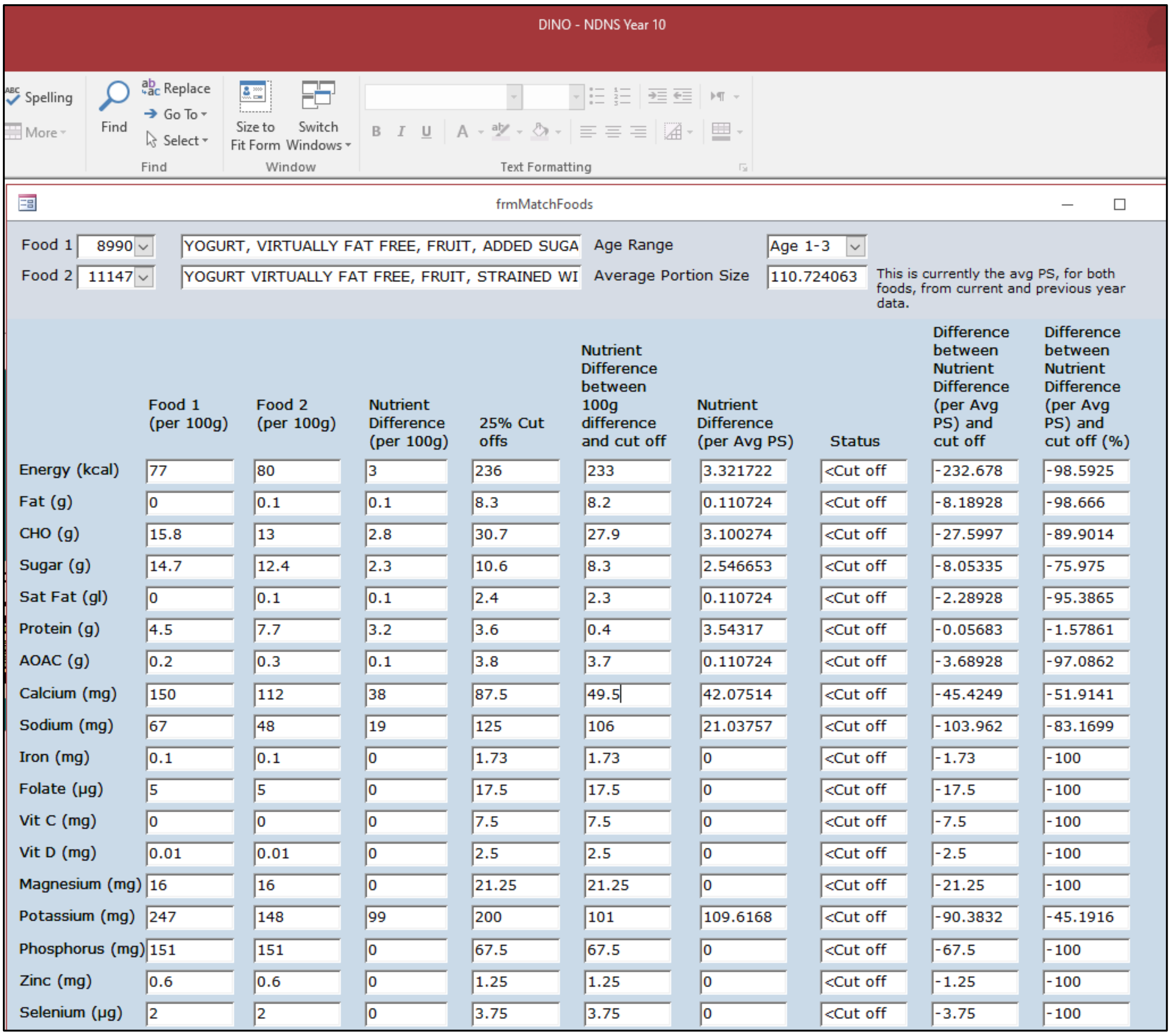

Food 1	8990	YOGURT, VIRTUALLY FAT FREE, FRUIT, ADDED SUGA	Age Range: Age 1-3
Food 2	11147	YOGURT VIRTUALLY FAT FREE, FRUIT, STRAINED WI	Average Portion Size: 110.724063

This is currently the avg PS, for both foods, from current and previous year data.

	Food 1 (per 100g)	Food 2 (per 100g)	Nutrient Difference (per 100g)	25% Cut offs	Nutrient Difference between 100g difference and cut off	Nutrient Difference (per Avg PS)	Status	Difference between Nutrient Difference (per Avg PS) and cut off	Difference between Nutrient Difference (per Avg PS) and cut off (%)
Energy (kcal)	77	80	3	236	233	3.321722	<Cut off	-232.678	-98.5925
Fat (g)	0	0.1	0.1	8.3	8.2	0.110724	<Cut off	-8.18928	-98.666
CHO (g)	15.8	13	2.8	30.7	27.9	3.100274	<Cut off	-27.5997	-89.9014
Sugar (g)	14.7	12.4	2.3	10.6	8.3	2.546653	<Cut off	-8.05335	-75.975
Sat Fat (gl)	0	0.1	0.1	2.4	2.3	0.110724	<Cut off	-2.28928	-95.3865
Protein (g)	4.5	7.7	3.2	3.6	0.4	3.54317	<Cut off	-0.05683	-1.57861
AOAC (g)	0.2	0.3	0.1	3.8	3.7	0.110724	<Cut off	-3.68928	-97.0862
Calcium (mg)	150	112	38	87.5	49.5	42.07514	<Cut off	-45.4249	-51.9141
Sodium (mg)	67	48	19	125	106	21.03757	<Cut off	-103.962	-83.1699
Iron (mg)	0.1	0.1	0	1.73	1.73	0	<Cut off	-1.73	-100
Folate (µg)	5	5	0	17.5	17.5	0	<Cut off	-17.5	-100
Vit C (mg)	0	0	0	7.5	7.5	0	<Cut off	-7.5	-100
Vit D (mg)	0.01	0.01	0	2.5	2.5	0	<Cut off	-2.5	-100
Magnesium (mg)	16	16	0	21.25	21.25	0	<Cut off	-21.25	-100
Potassium (mg)	247	148	99	200	101	109.6168	<Cut off	-90.3832	-45.1916
Phosphorus (mg)	151	151	0	67.5	67.5	0	<Cut off	-67.5	-100
Zinc (mg)	0.6	0.6	0	1.25	1.25	0	<Cut off	-1.25	-100
Selenium (µg)	2	2	0	3.75	3.75	0	<Cut off	-3.75	-100

References

1. Department of Health Committee on the Medical Aspects of Food Policy. *Dietary Reference Values for Food Energy and Nutrients for the United Kingdom*; London, UK, 1991.
2. Fitt, E.; Cole, D.; Ziauddeen, N.; Pell, D.; Stickley, E.; Harvey, A.; Stephen, A.M. DINO (Diet In Nutrients Out) - an integrated dietary assessment system. *Public Health Nutr* **2015**, *18*, 234-241, doi:10.1017/s1368980014000342.
