# Supplementary material for: Rationalisation of the UK Nutrient Databank for Incorporation in a Web-Based Dietary Recall for Implementation in the UK National Diet and Nutrition Survey Rolling Programme
Source: Nutrients. 2022 Oct 28;14(21):4551. doi: 10.3390/nu14214551 (PMC9658736; doi:10.3390/nu14214551)
Supplement: Supplementary file 1 [file nutrients-14-04551-s001.zip › Supplementary material-File S3.pdf]

File S3. Matching sandwiches reported in previous NDNS RP with food codes in the rationalised NDB.

In the NDNS year 2017 dataset, components of sandwiches i.e. bread, butter, cheese had not been linked together. Due to a large number of potential sandwiches, the following steps were undertaken to identify and relink the sandwiches to generic sandwich codes. An example of sandwich matching exercise is shown in Table S3a.

- Bread eaten with other foods consumed at the same time slot were collected in a database, there were 4,915 cases
- Removed the items that wouldn't consume in a sandwich such as tea; the list was reduced to 4,250 cases
- For efficiency, the food groups of ingredients were combined. There were 1,822 combinations e.g. bread; cheese; spread.
- More foods were excluded such as combinations with two ingredients or combinations including only bread and spreads (e.g. fat spread and jam).
- It was assumed that most sandwiches would have less than six ingredients, therefore sandwiches containing more than six ingredients were excluded.
- There were in total 1,826 combinations, and 1,070 of these were matched to a food code, using approximately 60 sandwich codes.
- As part of quality assessment, randomly selected matches were compared to original diary recordings. Various other checks were done such as the replacements where the difference in energy was highest, the respondents with the highest sandwich replacements, sandwiches consumed in odd amounts such as 2.5g.

Table S3a. Matching sandwiches, example tuna sandwich.[1]

List of combinations in NDNS year 2017 dataset	Number of ingredients	Number of consumption	Matched food code in rationalised NDB
Brown granary and wheatgerm bread : Manufactured canned tuna products incl ready meals : Butter :	3	6	FC 11427-Tuna mayo sandwich with wholemeal/oatmeal bread
Brown granary and wheatgerm bread : Manufactured oily fish products incl ready meals : Butter :	3	3	
Brown granary and wheatgerm bread : Manufactured oily fish products incl ready meals : Reduced fat spread (polyunsaturated)	3	3	
Wholemeal bread : Manufactured canned tuna products incl ready meals : Butter :	3	12	
Wholemeal bread : Manufactured canned tuna products incl ready meals : Savoury sauces pickles gravies & condiments :	3	3	

FC: Food code, [1]There were five combinations of three ingredients recorded at the same time slot, included bread and other ingredients. The listed combinations suggested that they could be a sandwich and according to the combination, they were matched to a most suitable sandwich.
