# Supplementary material for: Rationalisation of the UK Nutrient Databank for Incorporation in a Web-Based Dietary Recall for Implementation in the UK National Diet and Nutrition Survey Rolling Programme
Source: Nutrients. 2022 Oct 28;14(21):4551. doi: 10.3390/nu14214551 (PMC9658736; doi:10.3390/nu14214551)
Supplement: Supplementary file 1 [file nutrients-14-04551-s001.zip › Supplementary material-File S4.pdf]

File S4. Examples of main issues identified through systematic checks process.

Example 1. The “beef stew” which originally included meat and vegetables was matched to “stewed beef” which was 100% meat.

Example 2. Some breakfast cereals which included 100% cereal (e.g. Weetabix) were matched to a food code that included cereal and milk (e.g. porridge).

Example 3. The fruit content in smoothies doesn’t add to fruit intake in NDNS. Some of the smoothies reported in original year 10 are coded as multiple ingredients such as raw fruits and vegetables which look like 100% consumption of fruits in data. Whereas, when they were matched to a smoothie code in NDB the fruit amount was lower.
